# Hydro­thermally synthesized α-Ba_2_P_2_O_7_

**DOI:** 10.1107/S1600536810043527

**Published:** 2010-11-24

**Authors:** Carla Heyward, Matthew Mann, Joseph Kolis

**Affiliations:** a Department of Chemistry, Clemson University, Clemson, SC 29634, USA

## Abstract

Single crystals of α-Ba_2_P_2_O_7_, dibarium diphosphate, were obtained under hydro­thermal conditions. The structure belongs to the diphosphate *A*_2_P_2_O_7_ series with *A* being an alkaline earth cation. α-Ba_2_P_2_O_7_ crystallizes isotypically with α-Sr_2_P_2_O_7_. All atomic sites have site symmetry *m* with the exception of two O atoms which reside on general positions. Both Ba^2+^ cations are coordinated by nine terminal O atoms from eclipsed diphosphate P_2_O_7_ anions to form a three-dimensional network throughout the structure.

## Related literature

For general background, see: Brown & Calvo (1970 ▶); ElBelghitti *et al.* (1995 ▶); Mehdi *et al.* (1977 ▶); Mohri (2000 ▶). For the uses of alkaline earth diphosphates, see: McKeag & Steward (1955 ▶); Ranby *et al.* (1955 ▶); Ropp & Mooney (1960 ▶); Srivastava *et al.* (2003 ▶). For structurally related compounds, see: Calvo (1968 ▶); Barbier & Echard (1998 ▶). For an independent refinement of the α-Ba_2_P_2_O_7_ structure based on data from a crystal grown by solid-state reactions, see: Zakaria *et al.* (2010 ▶).

## Experimental

### 

#### Crystal data

Ba_2_P_2_O_7_
*M* *_r_* = 448.62
Orthorhombic, 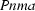

*a* = 9.2842 (19) Å
*b* = 5.6113 (11) Å
*c* = 13.796 (3) Å
*V* = 718.7 (3) Å^3^
*Z* = 4
Mo *K*α radiation
μ = 11.32 mm^−1^
*T* = 298 K
0.45 × 0.15 × 0.15 mm

#### Data collection

Rigaku AFC-8S Mercury CCD diffractometer
Absorption correction: multi-scan (*REQAB*; Jacobson, 1998 ▶) *T*_min_ = 0.080, *T*_max_ = 0.281
7205 measured reflections
1854 independent reflections
1510 reflections with *I* > 2σ(*I*)
*R*_int_ = 0.042

#### Refinement

*R*[*F* ^2^ > 2σ(*F* ^2^)] = 0.047
*wR*(*F* ^2^) = 0.127
*S* = 1.11
1854 reflections
58 parameters
Δρ_max_ = 6.71 e Å^−3^
Δρ_min_ = −3.53 e Å^−3^

### 

Data collection: *CrystalClear* (Rigaku/MSC, 2001 ▶); cell refinement: *CrystalClear*; data reduction: *CrystalClear*; program(s) used to solve structure: *SHELXTL* (Sheldrick, 2008 ▶); program(s) used to refine structure: *SHELXTL*; molecular graphics: *DIAMOND* (Brandenburg, 1999 ▶); software used to prepare material for publication: *SHELXTL*.

## Supplementary Material

Crystal structure: contains datablocks I, global. DOI: 10.1107/S1600536810043527/wm2406sup1.cif

Structure factors: contains datablocks I. DOI: 10.1107/S1600536810043527/wm2406Isup2.hkl

Additional supplementary materials:  crystallographic information; 3D view; checkCIF report

## Figures and Tables

**Table:** 

Ba1—O5^i^	2.564 (6)
Ba1—O1^ii^	2.730 (4)
Ba1—O4^iii^	2.799 (4)
Ba1—O1	2.903 (4)
Ba1—O2^iv^	2.9272 (18)
Ba2—O1^iv^	2.765 (4)
Ba2—O4^ii^	2.767 (4)
Ba2—O2^v^	2.800 (5)
Ba2—O4^vi^	2.836 (4)
Ba2—O5^vii^	3.084 (3)
P1—O1	1.514 (5)
P1—O1^viii^	1.514 (5)
P1—O2	1.519 (6)
P1—O3	1.588 (6)
P2—O4	1.515 (4)
P2—O4^viii^	1.515 (4)
P2—O5	1.519 (6)
P2—O3	1.598 (5)

**Table:** 

P1—O3—P2	134.7 (4)

Symmetry codes: (i) 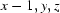; (ii) 
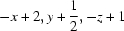
; (iii) 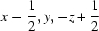; (iv) 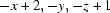; (v) 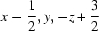; (vi) 
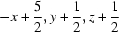
; (vii) 
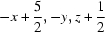
; (viii) 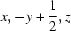.

**Fig. 1.**
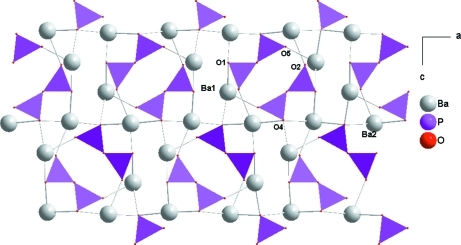
A view of the α-Ba2P2O7 structure along the b axis. Some Ba—O bonds are omitted for clarity. The purple polyhedra represent the diphosphate P2O7 groups.

**Fig. 2.**
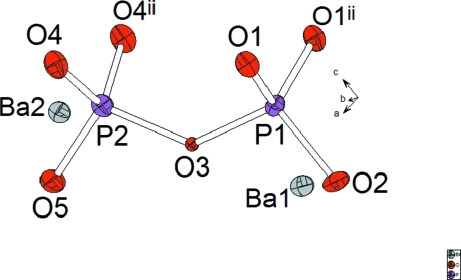
A view of the asymmetric unit of α-Ba2P2O7 with additional symmetry-related atoms displayed to show the tetrahedral environment around phosphorus. The Ba—O bonds were left out for clarity. Displacement ellipsoids are drawn at the 50% probability level. [Symmetry codes: (ii) x, -y + 1/2, z].

## supplementary crystallographic information

### Comment

Traditionally, alkaline earth diphosphates (or pyrophosphates) have been of interest as phosphor matrices in fluorescent lamps, among other applications (McKeag & Steward, 1955; Ranby *et al.*, 1955; Ropp & Mooney, 1960). More recently, research is focused on using these phosphor materials for multi-colored white LED devices (Srivastava *et al.*, 2003) to eliminate the use of mercury in fluorescent lamps. The activators ranged from metals such as manganese and tin to rare-earth elements like europium.

In the diphosphate *A*_2_P_2_O_7_ series (*A* = alkaline earth metal), when the ionic radius of *A* is greater than 0.97 Å, the structure is of the dichromate type and the P_2_O_7_ anion is in the eclipsed conformation as shown in Fig. 1 (Brown & Calvo, 1970). These structures include α-Ca_2_P_2_O_7_ which crystallizes in the monoclinic space group *P*2_1_/*n* (Calvo, 1968) and α-Sr_2_P_2_O_7_ in the orthorhombic space group *Pnma* (Barbier & Echard, 1998). In the case of barium, a hexagonal high-temperature form σ and an orthorhombic low-temperature form α are known to exist (ElBelghitti *et al.*, 1995). The structure of σ-Ba_2_P_2_O_7_ has been previously characterized from single crystal data in space group *P*62*m* and reported to have a very different structure from other known alkaline earth *A*_2_P_2_O_7_ diphosphates (ElBelghitti *et al.*, 1995). The more stable and common form α-Ba_2_P_2_O_7_ has been characterized by X-ray powder diffraction (Mehdi *et al.*, 1977), but there has been no previous report of the crystal structure determined from single-crystal data therefore, it is presented here. The cell parameters from our single-crystal data are in agreement with those of the previous powder diffraction study. The α nomenclature has been used for the orthorhombic Ba_2_P_2_O_7_ structure in previous reports (Mehdi *et al.*, 1977; Ranby *et al.*, 1955), consistent with being isostructural with α-Sr_2_P_2_O_7_ (Barbier & Echard, 1998).

The α-Ba_2_P_2_O_7_ structure contains two unique Ba atoms each surrounded by nine terminal oxygen atoms from diphosphate groups. The Ba1—O bond lengths range from 2.564 (6) - 2.9272 (18) Å and Ba2—O range from 2.765 (4) - 3.084 (3) Å. The Ba2 atoms connect to O4 along the *a*-axis to give a layered pattern. Ba1 atoms lie in between the layers and connect to O1 along the *c* axis resembling a ladder housing two eclipsed P_2_O_7_ groups in each section. Additional criss-cross action takes place with Ba1 connected to a terminal O5 atom and Ba2 connected to a terminal O2 atom of the diphosphate anion (Fig. 1). As mentioned before, α-Ba_2_P_2_O_7_ is isostructural with α-Sr_2_P_2_O_7_, whereas the α-Ca_2_P_2_O_7_ structure differs slightly. In this structure, the eight-coordinate Ca^2+^ cation is edge-sharing three O atoms and corner-sharing two O atoms with the P_2_O_7_ groups (Calvo, 1968). In the α-Ba_2_P_2_O_7_ and α-Sr_2_P_2_O_7_ structures, the nine-coordinate cations are edge-sharing four O atoms and corner-sharing one oxygen with the P_2_O_7_ groups (Barbier & Echard, 1998). The diphosphate group consists of two tetrahedral PO_4_ groups sharing O3 to form the P_2_O_7_ moiety (Fig. 2). A typical P—O bond length for the tetrahedral PO_4_ group is reported as 1.538 Å (Mohri, 2000) and the average terminal P—O bond distance in the title structure is 1.516 Å which is comparable to the average of 1.521 Å observed in both α-Sr_2_P_2_O_7_ (Barbier & Echard, 1998) and α-Ca_2_P_2_O_7_ (Calvo, 1968). In diphosphate groups, the bridging P—O bonds are characteristically longer. In the titled structure, the bridging P1—O3 and P2—O3 bonds are 1.588 (6) Å and 1.598 (5) Å which is in comparison to 1.579 (8) and 1.616 (8) Å reported for α-Ca_2_P_2_O_7_ (Calvo, 1968) and 1.599 (2) and 1.615 (2) Å observed in α-Sr_2_P_2_O_7_ (Barbier & Echard, 1998). The bridging O3 atom has longer bonds to P1 and P2. The P1—O3—P2 angle of 134.7 (4)° is wider than 130 (4)° reported for both α-Sr_2_P_2_O_7_ (Barbier & Echard, 1998) and α-Ca_2_P_2_O_7_ (Calvo, 1968) to help reduce structural strain resulting from the larger Ba^2+^ cation.

An independent refinement of the α-Ba_2_P_2_O_7_ structure based on data from a crystal grown by solid state reactions has been reported by Zakaria *et al.* (2010). The results of both refinements in terms of geometric parameters are the same within the threefold standard deviation.

### Experimental

The crystals were synthesized by combining 0.17 g of BaHPO_4_ and 0.05 g of NH_4_H_2_PO_4_ with 0.4 mL of 1*M* Ba(OH)_2_ solution in a sealed silver ampoule for 7–10 days at 773 K with a counter pressure of 19000 psi (131 MPa). The contents of the ampoule were washed with deionized water. Colorless needle shaped single crystals of α-Ba_2_P_2_O_7_ were the minor product and colorless polyhedrally shaped crystals of BaHPO_4_ were the major product.

### Refinement

Despite multiple data collections to high 2*θ* angles, the bridging oxygen atom O3 of the diphosphate group always appeared as non-positive definite when refined anisotropically. Therefore, we have refined this atom isotropically. The highest remaining peak is located 0.37 Å away from O3 and the deepest hole is 0.58 Å away from Ba1. The large density arises from O3 being defined as isotropically. The *xyz* coordinates for Q1 are 1.34 0.25 0.41 and for Q2 are 1.43 0.25 0.42 which are close to O3 at 1.38 0.25 0.42. The Q peaks are 0.37 and 0.54 Å away from O3 instead of the heavier atom Ba.

### Crystal data

**Table:**

Ba_2_P_2_O_7_	*F*(000) = 792
*M**_r_* = 448.62	*D*_x_ = 4.146 Mg m^−^^3^
Orthorhombic, *P**n**m**a*	Mo *K*α radiation, λ = 0.71073 Å
Hall symbol: -P 2ac 2n	Cell parameters from 3690 reflections
*a* = 9.2842 (19) Å	θ = 2.7–36.3°
*b* = 5.6113 (11) Å	µ = 11.32 mm^−^^1^
*c* = 13.796 (3) Å	*T* = 298 K
*V* = 718.7 (3) Å^3^	Needle, colorless
*Z* = 4	0.45 × 0.15 × 0.15 mm

### Data collection

**Table:**

Rigaku AFC-8S Mercury CCD diffractometer	1854 independent reflections
Radiation source: sealed tube	1510 reflections with *I* > 2σ(*I*)
graphite	*R*_int_ = 0.042
Detector resolution: 14.6306 pixels mm^-1^	θ_max_ = 36.3°, θ_min_ = 2.6°
ω scans	*h* = −8→15
Absorption correction: multi-scan (*REQAB*; Jacobson, 1998)	*k* = −7→9
*T*_min_ = 0.080, *T*_max_ = 0.281	*l* = −21→22
7205 measured reflections	

### Refinement

**Table:**

Refinement on *F*^2^	0 restraints
Least-squares matrix: full	Primary atom site location: structure-invariant direct methods
*R*[*F*^2^ > 2σ(*F*^2^)] = 0.047	Secondary atom site location: difference Fourier map
*wR*(*F*^2^) = 0.127	*w* = 1/[σ^2^(*F*_o_^2^) + (0.0617*P*)^2^ + 4.9464*P*] where *P* = (*F*_o_^2^ + 2*F*_c_^2^)/3
*S* = 1.11	(Δ/σ)_max_ = 0.001
1854 reflections	Δρ_max_ = 6.71 e Å^−^^3^
58 parameters	Δρ_min_ = −3.53 e Å^−^^3^

### Special details

**Table:**

Geometry. All e.s.d.'s (except the e.s.d. in the dihedral angle between two l.s. planes) are estimated using the full covariance matrix. The cell e.s.d.'s are taken into account individually in the estimation of e.s.d.'s in distances, angles and torsion angles; correlations between e.s.d.'s in cell parameters are only used when they are defined by crystal symmetry. An approximate (isotropic) treatment of cell e.s.d.'s is used for estimating e.s.d.'s involving l.s. planes.
Refinement. Refinement of *F*^2^ against ALL reflections. The weighted *R*-factor *wR* and goodness of fit *S* are based on *F*^2^, conventional *R*-factors *R* are based on *F*, with *F* set to zero for negative *F*^2^. The threshold expression of *F*^2^ > σ(*F*^2^) is used only for calculating *R*-factors(gt) *etc*. and is not relevant to the choice of reflections for refinement. *R*-factors based on *F*^2^ are statistically about twice as large as those based on *F*, and *R*- factors based on ALL data will be even larger.

### Fractional atomic coordinates and isotropic or equivalent isotropic displacement parameters (Å^2^)

**Table:**

	*x*	*y*	*z*	*U*_iso_*/*U*_eq_	
Ba1	0.86006 (5)	0.2500	0.41750 (3)	0.01365 (12)	
Ba2	0.84160 (5)	0.2500	0.74453 (3)	0.01249 (12)	
P1	1.2179 (2)	0.2500	0.45777 (12)	0.0120 (3)	
P2	1.4529 (2)	0.2500	0.31494 (13)	0.0120 (3)	
O1	1.1427 (4)	0.0289 (8)	0.4204 (3)	0.0163 (8)	
O2	1.2271 (6)	0.2500	0.5677 (4)	0.0164 (10)	
O3	1.3792 (6)	0.2500	0.4196 (3)	0.0118 (9)*	
O4	1.4045 (4)	0.0278 (7)	0.2617 (3)	0.0154 (7)	
O5	1.6144 (7)	0.2500	0.3326 (4)	0.0230 (12)	

### Atomic displacement parameters (Å^2^)

**Table:**

	*U*^11^	*U*^22^	*U*^33^	*U*^12^	*U*^13^	*U*^23^
Ba1	0.0139 (2)	0.0148 (2)	0.0122 (2)	0.000	−0.00132 (12)	0.000
Ba2	0.01179 (19)	0.0128 (2)	0.01293 (18)	0.000	0.00048 (12)	0.000
P1	0.0126 (7)	0.0131 (8)	0.0103 (6)	0.000	0.0008 (5)	0.000
P2	0.0105 (7)	0.0119 (7)	0.0138 (7)	0.000	0.0002 (5)	0.000
O1	0.0170 (18)	0.0134 (19)	0.0187 (18)	−0.0009 (14)	0.0018 (12)	0.0001 (13)
O2	0.018 (2)	0.018 (3)	0.013 (2)	0.000	−0.0009 (17)	0.000
O4	0.0149 (17)	0.0108 (17)	0.0205 (15)	−0.0033 (13)	0.0000 (13)	−0.0010 (13)
O5	0.011 (2)	0.037 (3)	0.022 (2)	0.000	0.001 (2)	0.000

### Geometric parameters (Å, °)

**Table:**

Ba1—O5^i^	2.564 (6)	Ba2—O2^viii^	2.800 (5)
Ba1—O1^ii^	2.730 (4)	Ba2—O4^ix^	2.836 (4)
Ba1—O1^iii^	2.730 (4)	Ba2—O4^x^	2.836 (4)
Ba1—O4^iv^	2.799 (4)	Ba2—O5^xi^	3.084 (3)
Ba1—O4^v^	2.799 (4)	Ba2—O5^x^	3.084 (3)
Ba1—O1^vi^	2.903 (4)	P1—O1	1.514 (5)
Ba1—O1	2.903 (4)	P1—O1^vi^	1.514 (5)
Ba1—O2^iii^	2.9272 (18)	P1—O2	1.519 (6)
Ba1—O2^vii^	2.9272 (18)	P1—O3	1.588 (6)
Ba1—P2^iv^	3.320 (2)	P1—Ba1^vii^	3.3700 (11)
Ba1—P1	3.369 (2)	P1—Ba1^iii^	3.3700 (11)
Ba1—P1^vii^	3.3700 (11)	P2—O4	1.515 (4)
Ba2—O1^iii^	2.765 (4)	P2—O4^vi^	1.515 (4)
Ba2—O1^ii^	2.765 (4)	P2—O5	1.519 (6)
Ba2—O4^iii^	2.767 (4)	P2—O3	1.598 (5)
Ba2—O4^ii^	2.767 (4)		
			
O5^i^—Ba1—O1^ii^	111.47 (14)	O2^viii^—Ba2—O4^x^	103.79 (13)
O5^i^—Ba1—O1^iii^	111.47 (14)	O4^ix^—Ba2—O4^x^	66.70 (16)
O1^ii^—Ba1—O1^iii^	69.95 (18)	O1^iii^—Ba2—O5^xi^	146.29 (14)
O5^i^—Ba1—O4^iv^	74.19 (15)	O1^ii^—Ba2—O5^xi^	78.59 (14)
O1^ii^—Ba1—O4^iv^	168.60 (12)	O4^iii^—Ba2—O5^xi^	129.35 (14)
O1^iii^—Ba1—O4^iv^	118.01 (12)	O4^ii^—Ba2—O5^xi^	66.99 (14)
O5^i^—Ba1—O4^v^	74.19 (15)	O2^viii^—Ba2—O5^xi^	71.70 (11)
O1^ii^—Ba1—O4^v^	118.01 (13)	O4^ix^—Ba2—O5^xi^	49.98 (14)
O1^iii^—Ba1—O4^v^	168.60 (12)	O4^x^—Ba2—O5^xi^	110.92 (14)
O4^iv^—Ba1—O4^v^	52.90 (17)	O1^iii^—Ba2—O5^x^	78.59 (14)
O5^i^—Ba1—O1^vi^	144.13 (12)	O1^ii^—Ba2—O5^x^	146.29 (14)
O1^ii^—Ba1—O1^vi^	75.66 (12)	O4^iii^—Ba2—O5^x^	66.99 (14)
O1^iii^—Ba1—O1^vi^	104.02 (8)	O4^ii^—Ba2—O5^x^	129.35 (14)
O4^iv^—Ba1—O1^vi^	93.97 (11)	O2^viii^—Ba2—O5^x^	71.70 (11)
O4^v^—Ba1—O1^vi^	71.85 (11)	O4^ix^—Ba2—O5^x^	110.92 (14)
O5^i^—Ba1—O1	144.13 (12)	O4^x^—Ba2—O5^x^	49.98 (14)
O1^ii^—Ba1—O1	104.02 (8)	O5^xi^—Ba2—O5^x^	130.9 (2)
O1^iii^—Ba1—O1	75.66 (12)	O1—P1—O1^vi^	110.0 (3)
O4^iv^—Ba1—O1	71.85 (11)	O1—P1—O2	111.48 (19)
O4^v^—Ba1—O1	93.97 (11)	O1^vi^—P1—O2	111.48 (19)
O1^vi^—Ba1—O1	50.61 (18)	O1—P1—O3	108.79 (19)
O5^i^—Ba1—O2^iii^	77.64 (11)	O1^vi^—P1—O3	108.79 (19)
O1^ii^—Ba1—O2^iii^	119.32 (14)	O2—P1—O3	106.1 (3)
O1^iii^—Ba1—O2^iii^	52.47 (14)	O4—P2—O4^vi^	110.7 (3)
O4^iv^—Ba1—O2^iii^	71.07 (13)	O4—P2—O5	111.7 (2)
O4^v^—Ba1—O2^iii^	121.96 (13)	O4^vi^—P2—O5	111.7 (2)
O1^vi^—Ba1—O2^iii^	131.20 (15)	O4—P2—O3	108.14 (19)
O1—Ba1—O2^iii^	80.75 (14)	O4^vi^—P2—O3	108.14 (19)
O5^i^—Ba1—O2^vii^	77.64 (11)	O5—P2—O3	106.1 (3)
O1^ii^—Ba1—O2^vii^	52.47 (14)	P1—O1—Ba1^iii^	101.23 (18)
O1^iii^—Ba1—O2^vii^	119.32 (14)	P1—O1—Ba2^iii^	136.0 (2)
O4^iv^—Ba1—O2^vii^	121.96 (13)	Ba1^iii^—O1—Ba2^iii^	110.49 (16)
O4^v^—Ba1—O2^vii^	71.07 (13)	P1—O1—Ba1	94.1 (2)
O1^vi^—Ba1—O2^vii^	80.75 (14)	Ba1^iii^—O1—Ba1	104.34 (12)
O1—Ba1—O2^vii^	131.20 (15)	Ba2^iii^—O1—Ba1	106.17 (13)
O2^iii^—Ba1—O2^vii^	146.9 (2)	P1—O2—Ba2^xii^	160.9 (4)
O1^iii^—Ba2—O1^ii^	68.95 (18)	P1—O2—Ba1^iii^	93.11 (12)
O1^iii^—Ba2—O4^iii^	72.50 (12)	Ba2^xii^—O2—Ba1^iii^	92.31 (11)
O1^ii^—Ba2—O4^iii^	109.70 (12)	P1—O2—Ba1^vii^	93.11 (12)
O1^iii^—Ba2—O4^ii^	109.70 (12)	Ba2^xii^—O2—Ba1^vii^	92.31 (11)
O1^ii^—Ba2—O4^ii^	72.50 (12)	Ba1^iii^—O2—Ba1^vii^	146.9 (2)
O4^iii^—Ba2—O4^ii^	68.58 (18)	P1—O3—P2	134.7 (4)
O1^iii^—Ba2—O2^viii^	141.37 (10)	P2—O4—Ba2^iii^	136.3 (2)
O1^ii^—Ba2—O2^viii^	141.37 (10)	P2—O4—Ba1^xiii^	96.06 (19)
O4^iii^—Ba2—O2^viii^	73.45 (13)	Ba2^iii^—O4—Ba1^xiii^	95.85 (12)
O4^ii^—Ba2—O2^viii^	73.45 (13)	P2—O4—Ba2^xiv^	104.2 (2)
O1^iii^—Ba2—O4^ix^	109.63 (12)	Ba2^iii^—O4—Ba2^xiv^	111.96 (14)
O1^ii^—Ba2—O4^ix^	73.36 (12)	Ba1^xiii^—O4—Ba2^xiv^	107.08 (13)
O4^iii^—Ba2—O4^ix^	176.88 (2)	P2—O5—Ba1^xv^	162.0 (4)
O4^ii^—Ba2—O4^ix^	112.28 (14)	P2—O5—Ba2^xiv^	93.88 (15)
O2^viii^—Ba2—O4^ix^	103.79 (13)	Ba1^xv^—O5—Ba2^xiv^	93.56 (13)
O1^iii^—Ba2—O4^x^	73.36 (12)	P2—O5—Ba2^xvi^	93.88 (15)
O1^ii^—Ba2—O4^x^	109.63 (12)	Ba1^xv^—O5—Ba2^xvi^	93.56 (13)
O4^iii^—Ba2—O4^x^	112.28 (14)	Ba2^xiv^—O5—Ba2^xvi^	130.9 (2)
O4^ii^—Ba2—O4^x^	176.88 (2)		

Symmetry codes: (i) *x*−1, *y*, *z*; (ii) −*x*+2, *y*+1/2, −*z*+1; (iii) −*x*+2, −*y*, −*z*+1; (iv) *x*−1/2, *y*, −*z*+1/2; (v) *x*−1/2, −*y*+1/2, −*z*+1/2; (vi) *x*, −*y*+1/2, *z*; (vii) −*x*+2, −*y*+1, −*z*+1; (viii) *x*−1/2, *y*, −*z*+3/2; (ix) −*x*+5/2, *y*+1/2, *z*+1/2; (x) −*x*+5/2, −*y*, *z*+1/2; (xi) −*x*+5/2, −*y*+1, *z*+1/2; (xii) *x*+1/2, *y*, −*z*+3/2; (xiii) *x*+1/2, *y*, −*z*+1/2; (xiv) −*x*+5/2, −*y*, *z*−1/2; (xv) *x*+1, *y*, *z*; (xvi) −*x*+5/2, −*y*+1, *z*−1/2.
